# MicroRNA-601 serves as a potential tumor suppressor in hepatocellular carcinoma by directly targeting PIK3R3

**DOI:** 10.3892/mmr.2022.12923

**Published:** 2022-12-22

**Authors:** Yanan Song, Saifei He, Juhua Zhuang, Guoyu Wang, Jing Ni, Suiliang Zhang, Ying Ye, Wei Xia

Mol Med Rep 19: 2431–2439, 2019; DOI: 10.3892/mmr.2019.9857

Following the publication of this paper, it was drawn to the Editors’ attention by a concerned reader that one of the data panels shown for the cell invasion assays in Fig. 2D was strikingly similar to another data panel that had appeared in different form in another article by different authors. Owing to the fact that the contentious data in the above article had already been published elsewhere prior to its submission to *Molecular Medicine Reports*, the Editor has decided that this paper should be retracted from the Journal. The authors were asked for an explanation to account for these concerns, but the Editorial Office did not receive a reply. The Editor apologizes to the readership for any inconvenience caused.

